# Meta-Transfer Learning Driven Tensor-Shot Detector for the Autonomous Localization and Recognition of Concealed Baggage Threats

**DOI:** 10.3390/s20226450

**Published:** 2020-11-12

**Authors:** Taimur Hassan, Muhammad Shafay, Samet Akçay, Salman Khan, Mohammed Bennamoun, Ernesto Damiani, Naoufel Werghi

**Affiliations:** 1 Center for Cyber-Physical Systems, Khalifa University of Science and Technology, Abu Dhabi 127788, UAE; 100057573@ku.ac.ae (M.S.); ernesto.damiani@ku.ac.ae (E.D.); naoufel.werghi@ku.ac.ae (N.W.); 2 Department of Computer Science, Durham University, Durham DH1 3DE, UK; samet.akcay@durham.ac.uk; 3 Inception Institute of Artificial Intelligence, Abu Dhabi 127788, UAE; salmaneme@gmail.com; 4 Department of Computer Science and Software Engineering, The University of Western Australia, Perth 6907, Australia; mohammed.bennamoun@uwa.edu.au

**Keywords:** aviation security, meta-transfer learning, one-shot learning, convolutional neural networks, structure tensors, X-ray imagery

## Abstract

Screening baggage against potential threats has become one of the prime aviation security concerns all over the world, where manual detection of prohibited items is a time-consuming and hectic process. Many researchers have developed autonomous systems to recognize baggage threats using security X-ray scans. However, all of these frameworks are vulnerable against screening cluttered and concealed contraband items. Furthermore, to the best of our knowledge, no framework possesses the capacity to recognize baggage threats across multiple scanner specifications without an explicit retraining process. To overcome this, we present a novel meta-transfer learning-driven tensor-shot detector that decomposes the candidate scan into dual-energy tensors and employs a meta-one-shot classification backbone to recognize and localize the cluttered baggage threats. In addition, the proposed detection framework can be well-generalized to multiple scanner specifications due to its capacity to generate object proposals from the unified tensor maps rather than diversified raw scans. We have rigorously evaluated the proposed tensor-shot detector on the publicly available SIXray and GDXray datasets (containing a cumulative of 1,067,381 grayscale and colored baggage X-ray scans). On the SIXray dataset, the proposed framework achieved a mean average precision (*mAP*) of 0.6457, and on the GDXray dataset, it achieved the precision and F_1_ score of 0.9441 and 0.9598, respectively. Furthermore, it outperforms state-of-the-art frameworks by 8.03% in terms of *mAP*, 1.49% in terms of precision, and 0.573% in terms of F_1_ on the SIXray and GDXray dataset, respectively.

## 1. Introduction

Baggage threat recognition has gained the utmost attention due to increased terrorist activities, especially in the last two decades. According to a recent survey, approximately 1.5 million passengers are screened every day against weaponry in the United States [1]. To identify baggage threats at the airport, malls, and cargoes, radiography is mainly used due to its reliability and cost-effectiveness [2]. In addition, many researchers have quantitatively measured the detection capacity of the security officers towards recognizing baggage threats through X-ray imagery via receiver operator characteristics (ROC) curve [3]. However, manual screening of baggage content (within the X-ray scans) to identify potential threats is a time-consuming task [4]. Furthermore, it is vulnerable to human errors caused due to fatigued work schedules [5]. Although, researchers have reported the high capacity (and less false alarm rate) of sniffer dogs to detect suspicious items as compared to humans. However, sniffer dogs can only work for an hour or so before they need rest [6]. Here, due to the capacity of autonomous frameworks to mass screen contraband items, many people have encouraged their utilization [4]. In addition, they recommended manual supervision (towards screening baggage threats) as a second-level inspection scheme to filter their erroneous detections [7].

**Figure 1:**
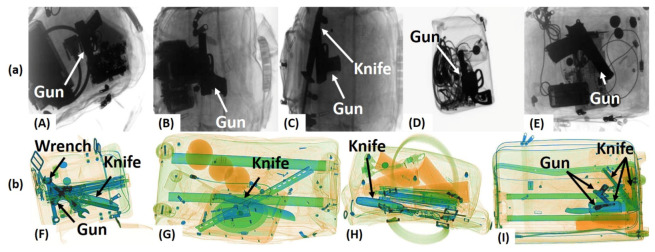
((**A**)–(**I**)) Baggage X-ray scans containing extremely cluttered and overlapping contraband items such as *guns*, *knives* and *wrenches*. Row (**a**) shows the scans from the GDXray [15] dataset and row (**b**) shows the scans from the SIXray [14] dataset.

For detecting objects from the RGB scans, many people have proposed one-staged and two-staged object detectors that produce promising results. However, due to the inherent differences between the X-ray and the RGB scans, these object detectors do not work well for identifying the baggage threats (via X-ray imagery) [8,9,10], especially in extreme concealment and cluttered scenarios [5,11]. To overcome this, researchers have developed exclusive frameworks for detecting and classifying baggage threats from the X-ray scans [12,13,14]. These frameworks can well recognize the visible and partially occluded baggage threats from the X-ray scans [5,11,13]. However, they are still vulnerable towards recognizing the extremely cluttered, concealed, and occluded objects [5,9] like, for example, the *guns* in Figure 1A–F,I, the *knives* in Figure 1F–I, and the *wrenches* in Figure 1F.

## 2. Related Work

Baggage threat detection has been a widely researched area where researchers initially employed conventional machine learning methods to recognize contraband items from the X-ray scan. Since the classical methods are based on hand-engineered features, they are confined to limited datasets and restricted experimental settings. More recently, deep learning has been employed for detecting prohibited items, outperforming traditional schemes in terms of accuracy, speed, and robustness. However, deep learning frameworks are still vulnerable to extreme occlusion, clutter, and diverse scanner specifications. Although, recent developments in recognizing baggage threats managed to address occlusion to some extent [5,13,14]. However, these frameworks are either tested on a single dataset [13,14] or they require extensive (parameter) tuning for different scanner specifications [5]. Furthermore, to the best of our knowledge, there is no mechanism (based on meta-learning [16] or meta-transfer learning [17]) to extend the capacity of these frameworks to generalize well across diverse ranging scanners without an explicit retraining process. In this section, we first shed light on some of the recent meta-learning (and meta-transfer learning [17]) frameworks, and then we discuss some of the popular frameworks for recognizing baggage threats. For an exhaustive survey on baggage threat recognition, we refer the readers to the work of [18,19,20].

### 2.1. Meta-Learning Frameworks

Meta-learning, also known as “learning to learn”, is a concept of extending the capacity of the deep neural networks to adapt (or generalize) to new tasks (or new domains) which have not been encountered during the training time. Essentially, the underlying network is given an exposure to learn from the large pool of experiences (during training), which they leverage on the set of unseen examples during the test time (via few-shot or zero-shot training). The major benefit of meta-learning over conventional transfer learning (or fine-tuning) approaches is that it allows the network to utilize its pretrained weights to effectively predict the unseen examples of the new underlying task without having to retrain on the large (and diverse) set of training examples for this current task to avoid overfitting [17]. Meta-learning has not only been employed for the supervised classification [16] and detection [21] tasks. It has also been used to acquire unlabeled data representation in an unsupervised manner [22]. More recently, Sun et al. [17] proposed a meta-transfer learning approach in which they transferred the pretrained weights of the deep neural networks for new tasks via few-shot learning where they achieved state-of-the-art performance on the benchmarked few-shot datasets such as miniImageNet [23] and Fewshot-CIFAR100 [24].

### 2.2. Traditional Machine Learning Methods

Initial solutions developed for baggage threat recognition involved classification [25], segmentation [26], and detection [27] strategies. While many of these schemes utilized SURF [28], and FAST-SURF [29] (coupled with Bag of Words), some of them also fused SIFT and SPIN features in conjunction with the Support Vector Machines (SVM) for classifying baggage threats from the multiview baggage imagery [8]. Moreover, Mery et al. proposed adaptive sparse representation [30] and adapted implicit shape model (AISM) [31] schemes for detecting prohibited baggage content. In another approach, they computed 3D feature points from the structure from motion to accurately recognize baggage threat from the X-ray imagery. In addition to this, Heitz et al. [26] proposed a region-growing technique coupled with SURF features to extract suspicious items from baggage X-ray scans.

### 2.3. Deep Learning Methods

Recently, researchers have developed deep learning methods for detecting prohibited items from the security X-ray scans. These methods have outperformed traditional approaches both in terms of robustness and efficiency. To increase readability, we have broadly categorized the deep learning methods (for screening baggage threats) as supervised and unsupervised approaches.

#### 2.3.1. Supervised Approaches

The supervised deep learning methods to recognize baggage threats are based on object detection [32,33,34], classification [35,36], and segmentation [11,37] schemes. The majority of these methods also utilize one-staged [38] and two-staged [9] detectors such as YOLO [39], YOLOv2 [40], RetinaNet [41], and Faster R-CNN [42]. Moreover, Akçay et al. used GoogleNet [43] for classifying contraband items such as *cameras*, *laptops*, *guns*, *gun components*, and *knives* (particularly *ceramic knives*) from their local X-ray dataset scans. Xiao et al. [44] developed a computationally efficient variant of Faster R-CNN [42] (namely R-PCNN) for detecting prohibited items from TeraHertz (THz) imagery. R-PCNN takes around 150 min on average for training and around 16 μs on average for detecting objects. Gaus et al. [10] evaluated RetinaNet [41], Faster R-CNN [42] and Mask R-CNN [45] backboned through ResNet-18 [46], ResNet-50 [46], ResNet-101 [46], SqueezeNet [47], and VGG-16 [48] for screening baggage X-ray scans as benign or malignant [10]. Griffin et al. [36] classified unexpected items within the bagging areas based upon their shape, texture, and density, and semantic appearances. Moreover, Dhiraj et al. [33] evaluated the Faster R-CNN [42], YOLOv2 [40] and Tiny YOLO [40] to detect contraband items such as *shuriken*, *guns*, *knives*, and *razors* from publicly available GRIMA X-ray Database (GDXray) [15]. Apart from this, Akçay et al. used AlexNet [49] as a features extractor coupled with SVM for baggage threat identification. Furthermore, they compared Faster R-CNN [42], sliding-window based CNN (SW-CNN), region-based fully convolutional networks (R-FCN) [50], and YOLOv2 [40] for recognizing occluded contraband items from the X-ray imagery. More recently, Wei et al. [13] proposed De-occlusion Attention Module (DOAM) module that can be integrated with the deep object detectors to recognize occluded threatening items. DOAM was thoroughly validated on a large-scale Occluded Prohibited Items X-ray (OPIXray) dataset, released publicly in [13]. Apart from this, Miao et al. [14] introduced one of the largest datasets for baggage threat detection, namely, Security Inspection X-ray (SIXray) dataset, containing extremely occluded and overlapping contraband items within highly imbalanced baggage X-ray scans. Furthermore, they proposed a framework, dubbed class-balanced hierarchical framework (CHR), to recognize contraband items such as *guns*, *knives*, *wrenches*, *pliers*, and *scissors* from different SIXray subsets indicating different levels of class imbalance [14]. SIXray dataset has been used by [51] in conjunction with their nonpublicly available dataset to analyze the transferability between different scanner specifications. Apart from this, we have also proposed a novel detection strategy, dubbed Cascaded Structure Tensor (CST), to recognize cluttered, occluded, and overlapping items from the SIXray [14] and GDXray [15] datasets.

#### 2.3.2. Unsupervised Approaches

The majority of baggage screening systems employ supervised strategies to recognize threatening items. However, researchers have also used unsupervised approaches (particularly adversarial learning) to recognize baggage threats as anomalies. Akçay et al. pioneered this by first proposing encoder-decoder-encoder topology coupled with adversarial learning, termed GANomaly [52]. Afterward, they employed skip-connections, yielding Skip-GANomaly [53], to derive better latent representations to aid discriminator in accurately picking the threatening anomalies [53].

As evident from Table 1, baggage threat detection is an extensively researched area where researchers have proposed different classification, detection, and segmentation approaches to recognize prohibited items from the security X-ray scans. These frameworks, though, can autonomously recognize the concealed contraband items under low or partial occlusion, but they are limited towards recognizing highly cluttered (and occluded objects). Recently, some researchers have proposed frameworks that address the problem of occlusion to some extent [5,13,14]. However, either these methods are tested on a single dataset [13,14] or they require a lot of parameter tuning due to nonadaptability [5]. Furthermore, to the best of our knowledge, all of the existing works require an extensive amount of training data (for each scanner specifications) to perform acceptable results. Procuring such large-scale data for training is not feasible, limiting the deployment of such frameworks in the real world.

## 3. Contributions

This paper presents a novel meta-transfer learning-driven tensor-shot detector that recognizes the baggage threats in extremely cluttered, concealed, and occluded environment. Furthermore, due to its capacity to operate on the unified tensor maps rather than diverse raw scans, it can be well-generalized across multiple scanner specifications via pretrained weights and single-shot learning. Moreover, we rigorously evaluated the proposed framework on two (highly challenging) public datasets where it achieves state-of-the-art performance. To summarize, the major contributions of this paper are thus four-fold:

- A novel meta-transfer learning based single-shot detector capable of recognizing baggage threats under extreme occlusion.
- A highly generalizable detection framework that leverages the proposed dual-tensor scheme to localize and recognize the threatening items from the diverse ranging scans without retraining the backbone on the large set of examples.
- To the best of our knowledge, there is no generalized framework that leverages meta-transfer learning to autonomously recognize concealed baggage threats from the joint (combined) GDXray [15] and SIXray [14] datasets.
- The proposed tensor-shot detector has outperformed state-of-the-art frameworks by achieving 1.49% and 0.573% improvements over [33] in terms of precision and F1 scores on GDXray [15] dataset and 8.03% improvements (in terms of mean average precision) over [14] on SIXray [14] dataset.

The rest of the paper is organized as follows: Section 4 presents the proposed tensor-shot framework. Section 5 contains a detailed description of the datasets, training, and evaluation protocols. Section 6 contains the experimental results and comparison of the proposed framework against state-of-the-art solutions and Section 7 presents a detailed discussion on the proposed framework and also concludes the paper.

## 4. Proposed Framework

**Figure 2:**
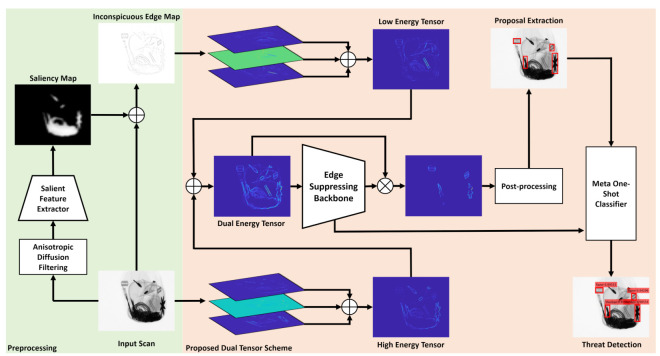
Block diagram of the proposed tensor-shot detector. First of all, we decompose the input scan into high and low energy tensors, where the high tensors are generated directly from the input scan (through the structure tensors [56]). Moreover, the low energy tensors are produced by first computing the salient image features (through the proposed feature extractor) and then accumulating them with the input scan. Both high and low energy tensors are then added together to produce a dual-energy tensor representation of the input scan that is then passed to the edge suppressing backbone to suppress the irrelevant baggage contours while simultaneously highlighting the threatening content. Then, the resultant baggage content is postprocessed, and for each extracted object, a bounding box is fitted to craft out its proposal that is passed to the meta-one-shot classifier for recognizing the object class.

The block diagram of the proposed detection framework is shown in Figure 2. It is driven through a novel dual tensor mechanism that exploits the transitional variations of baggage items (with diversified spatial properties) by simultaneously generating the low and high energy tensor representation of the candidate scan. These tensors are then accumulated together and are passed through the edge suppression backbone which filters the irrelevant edge information and only retains the boundaries of the potential threatening items. These filtered edges are then postprocessed, upon which the bounding boxes (screened through through nonmaximum suppression [55]) are fitted. Afterward, these bounding boxes are then used in crafting the object proposals which are further screened through the meta-one-shot classifier (driven through the edge suppression backbone).

### 4.1. Preprocessing

The input scan $\xi_{X}$ is filtered through the anisotrophic diffusion filter. Afterward, we generate the inconspicuous version of $\xi_{X}$ to enhance the edges of the dulled baggage items.

#### Inconspicuous Edge Map Generation

**Figure 3:**
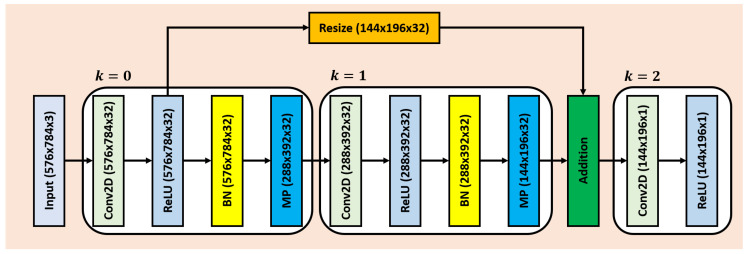
Salient network architecture. The abbreviations are Conv2D: 2D Convolution, BN: Batch Normalization, and MP: Max Pooling.

To generate the inconspicuous edge map, we first compute the saliency map (representing set of salient features) through the proposed salient feature extractor, and then eliminate these representations from the original input scan to highlight the edges of the low contrast and low spectral baggage items. The saliency map here showcases the items having the higher spectral components within $\xi_{X}$, derived through the trainable edge-preserving kernels of the proposed salient feature extractor. Moreover, the architecture of the feature extractor is intentionally kept shallow by deploying only one input layer, three convolution layers, two batch normalization layers, three ReLUs, two max pooling layer, one lambda layer (for resizing) and one addition layer (as shown in Figure 3) having a total of 1601 learnable and 128 nonlearnable parameters. The reason for making the salient network shallower is to preserve the shape of the prominent objects (having higher spectral components) that are eliminated from $\xi_{X}$ to retain the contours of the dulled items and also to avoid the generation of false edges.

From Figure 3, we can also observe that the proposed salient feature extractor contains three salient blocks denoted by k=0,1,2, wherein each block, the convolution and ReLU layers yields $f^{k}\left(x,y,z\right)$ and $f_{r}^{k}\left(x,y,z\right)$ of size $M_{1}\times M_{2}\times M_{3}$, respectively such that:

(1) $$f^{k}\left(x,y,z\right)=\sum_{i=1}^{N_{1}}\sum_{j=1}^{N_{2}}\sum_{h=1}^{N_{3}}w_{k}\left(i,j\right)f_{m}^{k}\left(\left[\frac{x-i+2p_{1}}{s_{1}}\right]+1,\left[\frac{x-j+2p_{2}}{s_{2}}\right]+1,\left[\frac{z-h+2p_{3}}{s_{3}}\right]+1\right)$$

and

(2) $$f_{r}^{k}\left(x,y,z\right)=\left\{\begin{array}{cc} f^{k}\left(x,y,z\right) & f^{k}\left(x,y,z\right)\geq 0\\ 0 & \mathrm{otherwise} \end{array}\right.$$

$w_{k}$ denotes the window of $N_{1}\times N_{2}\times N_{3}$ dimension (containing the trainable weights), $p_{1,2,3}$ denotes the input padding, $s_{1,2,3}$ denotes the stride rate, and $f_{m}^{k}$ denotes the input feature maps. It should be noted here that for k=0, $f_{m}^{0}\left(x,y,z\right)=\xi_{X}$, i.e., the input to the first convolution layer is the input scan $\xi_{X}$. Moreover, after extracting $f_{r}^{k}\left(x,y,z\right)$, it is normalized through the batch normalization layer as expressed below:

(3) $$g^{k}\left(x,y,z\right)=\sum_{i=1}^{M_{1}}\sum_{j=1}^{M_{2}}\sum_{h=1}^{M_{3}}\frac{f_{r}^{k}\left(i,j,h\right)-\mu \left(f_{r}^{k}\right)}{\sqrt{\sigma (f_{r}^{k})}}$$

where $\mu (f_{r}^{k})$ and $\sigma (f_{r}^{k})$ represent a mean and variance of the feature maps in *k*th block i.e., $f_{r}^{k}$, respectively. Then, $g^{k}\left(x,y,z\right)$ is passed through the max pooling layer, producing $g_{m}^{k}\left(x,y,z\right)$ such that:

(4) $$g_{m}^{k}\left(x,y,z\right)=\underset{\Delta_{x}\in \frac{-K_{1}}{2},...,\frac{K_{1}}{2},\Delta_{y}\in \frac{-K_{2}}{2},...,\frac{K_{2}}{2},\Delta_{z}\in \frac{-K_{3}}{2},...,\frac{K_{3}}{2}}{max}\;g^{k}\left(x+\Delta_{x},y+\Delta_{y},z+\Delta_{z}\right)$$

$K_{1}$, $K_{2}$ and $K_{3}$ here denotes the pooling dimensions and the operations described in Equations (1)–(3) are performed in a cascaded fashion for k=1,2 as well. However, for k=2, the input to the convolution layer is a fusion between resized high resolution features ($f_{r}^{0}{\left(x,y,z\right)}^{\prime }$), and the output of the previous salient block i.e., $f^{2}\left(x,y,z\right)=g_{m}^{1}\left(x,y,z\right)+f_{r}^{0}{\left(x,y,z\right)}^{\prime }$. In addition, the batch normalization and pooling operations are not performed at k=2, rather, the network outputs $f_{r}^{2}\left(x,y,z\right)$ as the salient features. Afterward, the inconspicuous edge map (generated by accumulating the saliency features $f_{r}^{2}\left(x,y,z\right)$ with the input scan) is decomposed into a low energy tensor, which is further added with its high energy counterpart to generate a dual-tensor map. Here, contrary to the recent data fusion approaches (which use additional thermal [57] or depth [58] encoders), our approach utilizes a single lightweight feature extractor (containing only 1729 parameters) to produce good salient feature representations as evident from Figure 6 in Section 6.

### 4.2. Proposed Dual Tensor Scheme

After obtaining the inconspicuous edge map, we decompose it (along with the original input scan) into the low and high energy tensors to reveal the transitional variations of all the baggage content irrespective of their spatial characteristics. The motivation for proposing the dual-energy tensors stems from the fact that objects within the baggage X-ray scans exhibit different spatial characteristics. Some are composed of higher spectral bands whereas others blend more with the background (see, for example, the *shuriken* and *razors* in Figure 2). Therefore, such objects cannot be picked in one-go (especially through the trivial edge detection and representation methods). The proposed dual-tensor scheme amplifies the transitional variations of the cluttered items as compared to the state-of-the-art methods [5], leading towards more robust identification of the cluttered baggage threats. This dual-tensor decomposition within the proposed framework is performed through structure tensor [56], which in its simplest form a 2×2 symmetric matrix computed (pixel-wise) by taking an outer product of image gradients (defined by the neighborhood of each pixel within the candidate scan) [56], as expressed in Equation (5).

(5) $${ℑ}_{S}=\left[\begin{array}{cc} \varphi *(\nabla X.\nabla X) & \varphi *(\nabla X.\nabla Y)\\ \varphi *(\nabla Y.\nabla X) & \varphi *(\nabla Y.\nabla Y) \end{array}\right]$$

where each outer product φ*(∇i.∇j), dubbed tensor, denotes the outer product of image gradients ∇i and ∇j oriented at direction *i* and *j*, respectively. φ denotes the Gaussian diffusion filter responsible for removing noisy outliers while retaining the transitional information of all the objects. It is computed through Equation (6):

(6) $$\varphi =\sum_{s\in \omega_{x}}\sum_{t\in \omega_{y}}\frac{1}{2\pi \sigma^{2}}e^{-\frac{s^{2}+t^{2}}{2\sigma^{2}}}I\left(i,j;k\right)$$

(7) $$I\left(i,j;k\right)=\sum_{n_{k}\in <\omega_{x},\omega_{y}>}\frac{1}{n_{k}!\Gamma \left(n_{k}+k+1\right)}{\left(\frac{i+j}{2}\right)}^{2n_{k}+k}$$

where I(i,j;k) denotes the modified Bessel function of *k*th order and Γ(.) represents the gamma function, i.e., Γ(k)=(k−1)!,∀k∉C. The block matrix in Equation (5) yields four outer products (tensors) from the candidate scan, where only three of them are unique (since this matrix is symmetric). Afterward, we add these tensors together to generate a single high energy tensor map (containing objects with the higher frequency components) and a single low energy tensor (depicting dulled baggage content). These low and high energy tensors are further added together to generate a dual-energy tensor representation of the candidate scan as shown in Figure 2.

### 4.3. Edge Suppressing Backbone

The dual-energy tensor map emphasizes the edge representation of the dulled contraband items while retaining the prominent features of the baggage scans. However, before fitting the bounding boxes to localize the threatening items, we pass the dual-tensor map through the edge suppression backbone, trained via meta-transfer learning [17], to strain the irrelevant boundaries of the normal baggage content while only preserving the edges of the threatening items. The choice of the backbone network is extensively discussed in the ablation study (Section 6.1). In addition, the training of the backbone network via meta-transfer learning is presented in Section 4.5. Moreover, the processed tensor (obtained by multiplying the dual-energy tensor with the output of the backbone network) is then binarized from which the bounding boxes are fitted to localize the contraband items. The duplicate and redundant bounding boxes are removed through the nonmaximum suppression [55]. Afterward, these bounding boxes are utilized in cropping the object proposals from $\xi_{X}$ which are then recognized via the meta-one-shot classifier.

### 4.4. Meta-One-Shot Classifier

Due to the capacity of the edge suppressing backbone network to differentiate between the contours of the threatening items and the normal baggage content, we also deploy it in conjunction with the fully connected layers to recognize the localized threatening items. Here, we fine-tune the backbone network (coupled with the fully connected layers) to recognize contraband items within the cropped object proposals via a single training example of each suspicious item category (i.e., we perform one-shot learning to recognize the proposals of the contraband items).

### 4.5. Training via Meta-Transfer Learning

In order to generalize the proposed tensor-shot detector to extract the contraband items irrespective of the scanner specifications, and also to avoid the requirement of large and well-annotated data for fine-tuning the pretrained weights of the backbone, we adopted meta-transfer learning [17] strategy as described in Algorithm 1. Here, ”task” refers to the correct identification of each suspicious item category, and the meta-transfer learning (for the proposed tensor-shot detector) is performed in an iterative manner where, in the first iteration, we trained the backbone model on the dual-energy tensors (obtained from the joint GDXray [15] and SIXray [14] datasets) to suppress the contours of normal baggage content while retaining the edges of the prohibited items. Moreover, in the second iteration, we take the model weights (θ) (updated in the first iteration) and fine-tune them through the single-shot training to classify the localized proposal categories. The network weights (θ) learned in the first iteration enables the network to effectively recognize the baggage items (within each proposal) without retraining the whole network again for each dataset separately. Even fine-tuning on the single example of each category (in the second iteration) is optional as the proposed detector also produces decent performance with the zero-shot classifier (please see Section 6.5 for more details). Apart from this, the complete implementation details of the proposed detection framework are presented in Section 5.2.

 **Algorithm 1:** Meta Transfer Learning Algorithm 
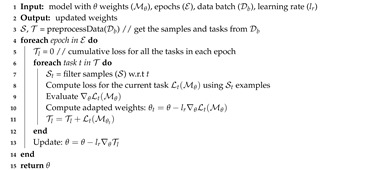


### 4.6. Loss Function

The dual-tensor map contains imbalanced ratio of normal and threatening items contours. Therefore, penalizing the backbone model through the conventional cross-entropy loss function would make it biased towards producing more false positives (and false negatives as well). Therefore, in order to effectively train the model to distinguish between normal and threatening baggage content, we employ a focal loss function [41] within the proposed tensor-shot framework, as expressed below:

(8) $$L=-\frac{1}{b_{s}}\sum_{i=0}^{b_{s}-1}\sum_{j=0}^{c-1}\alpha {\left(1-p\left(l_{i,j}\right)\right)}^{\gamma }t_{i,j}\log\left(p\left(l_{i,j}\right)\right)$$

where $b_{s}$ is the batch size, *c* denotes the total number of classes, $t_{i,j}$ indicates whether or not the *i*th training example is from the *j*th class, $p(l_{i,j})$ represents the probability of the logit $l_{i,j}$ (generated by the network) for the *i*th training example belonging to the *j*th class, the expression $\alpha {\left(1-p\left(l_{i,j}\right)\right)}^{\gamma }$ depicts the scaling factor [41]. The values for the focal loss parameters are determined empirically through rigorous experimentation, as reported in the ablation study (Section 6.1.1).

## 5. Experimental Setup

In this section, we present a detailed description of the datasets, the implementation details as well as the evaluation metrics, which we used to compare the proposed framework with the state-of-the-art solutions.

### 5.1. Datasets

The proposed framework has been extensively evaluated on publicly available GRIMA X-ray database (GDXray) [15] and Security Inspection X-ray (SIXray) [14] dataset. GDXray [15] is the widely used dataset containing high resolution texture-less grayscale X-ray scans [15]. Moreover, SIXray [14] is the recently introduced large-scale dataset for baggage threat detection and to date it contains the most challenging colored X-ray scans. The detailed description of each dataset is presented below:

#### 5.1.1. GRIMA X-ray Database

GDXray [15] is the widely used public dataset for baggage threat detection and also for the nondestructive testing (NDT) [15]. GDXray [15] is unique as it is the only public dataset containing the 19,407 texture-less grayscale scans in which it contains the baggage items that are heavily occluded as shown in Figure 1. Moreover, the scans within GDXray [15] are highly annotated and arranged within five categories, i.e., *welds*, *baggage*, *nature*, *casting*, and *settings*. The baggage groups (which is the only relevant category for this study) contain 8150 grayscale X-ray scans in which the suspicious items such as *handguns*, *razors*, *shuriken*, and *knives* have been marked by the security experts. Apart from this, we have marked the suspicious items in the original dataset (like *chip* and *mobile phones*) to further validate the performance of the proposed framework. To make things even more challenging, we have separated the original *handgun* category as *pistol* and *revolver*, to further test the capacity of the proposed detection framework in individually recognizing these items.

#### 5.1.2. Security Inspection X-ray Dataset

SIXray [14] is the largest and, to the best of our knowledge, the most challenging dataset for the extraction and identification of baggage items from the colored X-ray images [14]. The dataset contains 1,059,231 scans having heavily occluded and cluttered items in which the suspicious items are grouped into six categories, i.e., *knives*, *guns*, *wrenches*, *scissors*, *pliers*, and *hammers*. Furthermore, the dataset has been organized into various subsets containing a highly imbalanced combination of positive and negative scans (positive means scan having one or more suspicious item and negative means scan having no suspicious item) to meet the real-world scenario. These subsets are named as SIXray10, SIXray100, and SIXray1000, respectively [14]. Apart from this, the dataset contains highly detailed annotations of baggage items that were marked by the security experts. These annotations served as ground truth for us to validate the performance of the proposed framework.

Here, we further want to highlight that both of these datasets contain a wide range of forbidden items that have been identified by the European Commission in this report [59].

### 5.2. Implementation Details

The proposed detection framework has been implemented on MATLAB R2020a using the deep learning, computer vision, and image processing toolbox on a machine with Intel Core i5, 16 GB RAM, and NVIDIA RTX 2080 GPU (with compute compatibility v7.5). For a fair comparison with the existing solutions, the scans used for training and testing the proposed tensor-shot detector were honored as per each dataset standard. First of all, we trained the salient network for 5 epochs on each dataset. Afterward, we conducted meta-transfer learning for 10 epochs (in the first iteration) to generalize the backbone network in distinguishing the edge representation of the normal and threatening baggage content based upon the 848,172 dual-energy tensors obtained from the training scans of combined GDXray [15] and SIXray [14] datasets. Moreover, in the second iteration, the meta-transfer learning was conducted for 2 epochs in which we trained the meta-one-shot classifier (with a single training example of each contraband item proposal) to effectively recognize them. Apart from this, we employed the stochastic gradient descent as an optimizer with a momentum of 0.9 and a static learning rate ($l_{r}$) of 0.001. These hyperparameters are determined empirically for both datasets through conventional grid search optimizations [60,61], where the learning rate was varied from 0.1 to 0.0001 by the drop factor of $\frac{1}{10}$, and momentum was varied from 0.5 to 0.95 in the step of 0.05.

### 5.3. Evaluation Metrics

To evaluate the performance of the proposed framework and also to compare it with the state-of-the-art solution, we have used the following metrics:

#### 5.3.1. Intersection-over-Union

Intersection over Union (*IoU*), also known as Jaccard’s similarity index measures the capacity of the framework that how well it has extracted the object of interest as compared to its ground truth. The *IoU* is computed through [62]:

(9) $$IoU=\frac{T_{p}}{T_{p}+F_{p}+F_{n}}$$

where *T_p_* denotes the true positives, *F_p_* represents false positives, and *F_n_* denotes the false negatives. Moreover, the mean *IoU* score, showcasing the overall object extraction performance of the proposed framework, is computed by taking an average of *IoU* score for each suspicious item category.

#### 5.3.2. Dice Coefficient

Dice Coefficient (*DC*) also illustrates how accurately the proposed framework can extract the object regions and it is computed by measuring a degree of similarity between the extracted object regions with respect to their ground truths as expressed in Equation (10) [63]:

(10) $$DC=\frac{2T_{p}}{2T_{p}+F_{p}+F_{n}}$$

Moreover, the mean *DC* is computed by taking the average of *DC* scores for all the suspicious items categories. The difference between *IoU* and *DC* is that *DC* gives more weight towards the accurate extraction of the contraband items (true positives) as compared to the *IoU*.

#### 5.3.3. Mean Average Precision

The performance of the proposed framework for accurately detecting the prohibited items is measured by the mean average precision (*mAP*) scores. Here, the *mAP* scores are measured by taking the mean of average precision (*AP*) scores computed at an *IoU* ≥ 0.5 for each suspicious item category.

(11) $$mAP=\sum_{k=0}^{n_{c}-1}AP\left(k\right)$$

where *n_c_* denotes the total number of contraband item categories.

#### 5.3.4. Confusion Matrix

Apart from evaluating the detection performance of the proposed framework using *mAP*. We also validated its capacity to classify the baggage scan as threatening or nonthreatening using the confusion matrix and standard classification metrics such as accuracy, true positive rate (*TPR*), false positive rate (*FPR*), positive predicted value (*PPV*), and the *F_1_* score as expressed below:

(12) $$Accuracy=\frac{T_{p}+T_{n}}{T_{p}+F_{n}+F_{p}+T_{n}}$$

(13) $$TPR=\frac{T_{p}}{T_{p}+F_{n}}$$

(14) $$FPR=\frac{F_{p}}{F_{p}+T_{n}}$$

(15) $$PPV=\frac{T_{p}}{T_{p}+F_{p}}$$

(16) $$F_{1}=2\times \frac{TPR\times PPV}{TPR+PPV}$$

where *T_n_* denotes the true negatives.

#### 5.3.5. Mean Squared Error

To further show the statistical significance of the proposed framework compared to the state-of-the-art solutions on both GDXray [15] and SIXray [14] dataset. We have used the mean squared error (*MSE*) scores. *MSE*, in this study, is computed for each contraband item class through Equation (17) [64]:

(17) $$MSE=\frac{1}{n_{t}}\sum_{i=1}^{n_{t}}{\left(y_{i}-\hat{y_{i}}\right)}^{2}$$

where $y_{i}$ denotes the ground truth values for each item, $\hat{y_{i}}$ denotes the predicted values of each contraband item, and $n_{t}$ denotes the total number of instances of the respective item within the dataset. Here, it should be noted that the predicted values for each item are taken as their *mAP* scores, and their ground truth values represent ideal *mAP* performances, i.e., 1.

#### 5.3.6. Qualitative Evaluations

Apart from quantitative evaluations, we also demonstrate the capacity of the proposed framework for accurately detecting the cluttered, concealed, and overlapping contraband items through extensive qualitative examples.

## 6. Results

In this section, we present a thorough evaluation of the proposed framework on two publicly available datasets. Furthermore, we showcase its detailed comparison with the state-of-the-art frameworks against various metrics. We also present an ablation study here through which we determined the optimal parameters for the focal loss function [41] and the backbone model for detecting the baggage threats.

### 6.1. Ablation Study

Before discussing the experimental results of the proposed framework, we present an ablation study where we determined the optical parameters for the focal loss function [41] and best backbone network for edge suppression and object proposals classification.

#### 6.1.1. Determining the Focal Loss Parameters

The scaling factor within the focal loss function [41] consists of two hyperparameters, i.e., the α and the γ parameter. α represents the balancing factor that penalizes the network towards accurately recognizing the imbalanced classes, and γ is the focusing parameter that allows the network to down-weight the accurate recognition of easy examples to emphasize on the hard one. Here, we varied the value of α as 0.25≤α≤0.75, and the value of γ as 1≤γ≤5 for both GDXray [15] and SIXray [14] datasets according to the grid search scheme [60,61]. From Table 2, we can observe that for GDXray [15] dataset, varying the value of α and β does not affect much the overall detection performance of the proposed framework. This is because GDXray [15] does not contain highly imbalanced contraband item classes. However, on SIXray [14], we see significant variations in the detection performance while varying α and γ, i.e., we achieved the maximum *mAP* score of 0.6457 on SIXray [14] dataset with α=0.25,γ=2, and a minimum *mAP* score of 0.4926 with α=0.75,γ=1. Here, it should also be noted that increasing the value of γ penalizes the proposed framework to focus more on the hard examples, whereas decreasing the value of α ensures high resistance to the imbalanced classes.

#### 6.1.2. Determining the Classification Backbone

To determine the best backbone model, we tested the tensor-shot detector with ResNet-50 [46], ResNet-101 [46] and VGG-16 [48], where the detection performance with each of the backbones is reported in Table 3. We can observe here that although the best detection results are achieved with ResNet-101 [46] on both datasets, the choice of backbone does not significantly affect the overall detection performance of the proposed framework, e.g., the worse detection performance with VGG-16 [46] only lags by 5.14% on GDXray [15] and 5.83% on SIXray10 [14] from the best performing ResNet-101 [46] backbone.

### 6.2. Evaluations on GDXray Dataset

The first dataset on which we evaluated the proposed framework is the GDXray [15] dataset. The detection performance of the proposed framework on GDXray [15] is shown in Table 4. Here, we can observe that the proposed framework achieved the mean *IoU*, mean *DC* and the *mAP* score of 0.9118, 0.9536, and 0.9162, respectively. Furthermore, it outperformed [33] by achieving 1.49% improvements in terms of *PPV* and 0.573% improvements in terms of F_1_ score. However, it lags from [33] by 0.397% in terms of *TPR* and 2.90% in terms of accuracy. With that said, since F_1_ is a better score than accuracy especially for the imbalanced data and considering the fact that the proposed framework is also validated using standard *mAP* metric (where it achieved the score of 0.9162), we believe that the performance of the proposed framework is significant.

In addition to this, Table 5 reports the statistical analysis of the proposed framework in terms of *MSE* scores. Here, to make the fair comparison with state-of-the-art frameworks, we only extracted the originally marked contraband items from the dataset i.e., *handguns*, *knives*, *razors*, and *shuriken*. We can observe from Table 5 that the proposed framework statistically outperforms the second-best [5] by 40.05% that is quite significant, especially because the framework in [5] is a fully supervised framework trained via conventional fine-tuning. However, the proposed framework employs meta-transfer learning for detecting suspicious baggage items.

**Figure 4:**
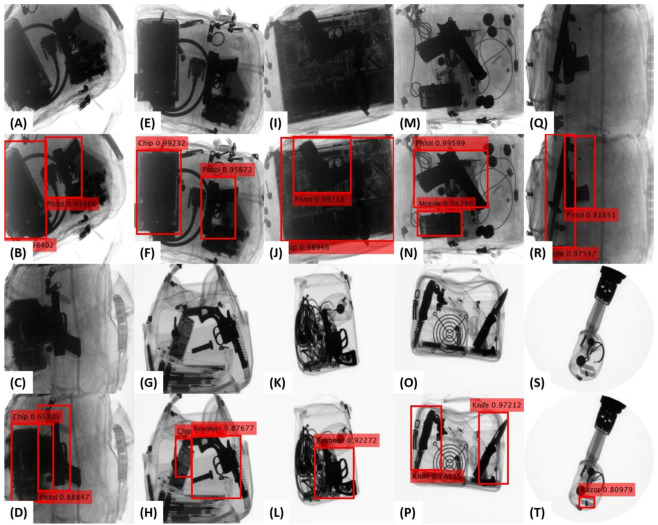
Qualitative performance of the proposed framework on the GDXray [15] dataset for detecting contraband items such as *pistols*, *revolvers*, *razors*, *chips*, *mobile phones*, and *knives* as shown in (**A**–**T**). Moreover, first and third row show the original scans. We can appreciate the detection of concealed *pistol* in (**B**,**F**), concealed *pistol* and a *laptop* (*chip*) in (**J**), the cluttered *pistol* and *knife* in (**R**), cluttered *revolver* in (**L**), and low contrasted *razor* in (**T**).

Apart from this, we also present the qualitative evaluation of the proposed detection framework in Figure 4 where we can observe how effectively the proposed tensor-shot detector recognizes the concealed and cluttered contraband items. For example, see the detection of concealed pistol in (B and F), concealed *pistol* and a *laptop* (*chip*) in (J), the cluttered *pistol* and *knife* in (R), cluttered *revolver* in (L), and low contrasted *razor* in (T).

### 6.3. Evaluations on SIXray Dataset

The second dataset on which we have evaluated the proposed framework is the SIXray [14] dataset. SIXray [14] to the best of our knowledge is the largest and most challenging baggage X-ray dataset to date [14]. The detection performance of the proposed framework can be seen in Table 4 where we can observe that the proposed detector achieved an *mAP* score of 0.6457, outperforming [14] by 8.03%. Although it lags from [51] by 24.03%. However, this comparison is not fair because the authors in [51] only utilized SIXray10 subset of the SIXray dataset for extracting only the *guns* and *knives*. However, we evaluated the proposed framework on all the three subsets of the SIXray [14] dataset for extracting all the originally marked prohibited items [14]. Apart from this, we achieved an F_1_ score of 0.1153 on SIXray [14] dataset. We can notice here the substantial gap of 87.11% between the performance of the proposed framework in terms of accuracy and the F_1_ score. This is due to the fact that all the subsets of SIXray [14] dataset are extremely imbalanced [14]; therefore, we got an excessive number of false positives compared to the true positives (causing a very low precision and F_1_ score).

In another experiment, we quantified the capacity of the proposed framework to detect contraband items under various degrees of clutter and concealment. For this, we divided the positive scans within SIXray [14] dataset into three disjoint sets. The first set contains only those examples which contain contraband items under the low concealment. The second set contains examples with partially cluttered suspicious objects, and the third set contains extremely cluttered and concealed contraband items. Please note that these sets are prepared by us just to give the quantitative representation on how well the proposed framework is resistant to the level of clutter, and we also want to highlight these sets are not present within the original SIXray [14] dataset. Furthermore, we performed this experiment only on the SIXray [14] dataset because SIXray [14] is, to the best of our knowledge, the largest and most challenging dataset designed for detecting baggage threats under the highly imbalanced scenario. GDXray [15], although, contains texture-less grayscale scans making the detection of contraband items (in some scans) difficult. However, overall, comparing the complexity of GDXray [15] with SIXray [14], SIXray [14] presents more challenging cases. The quantitative evaluation of the proposed framework for this experiment is shown in Table 6. Here, we can observe how effectively the proposed framework recognizes the suspicious items regardless of the clutter, occlusion, or concealment. Even in an extremely cluttered scenario, the performance of the proposed framework only deteriorates by 33.45%, which is 4.40% better than [14].

Moreover, Table 5 reports the statistical significance of the proposed framework in terms of *MSE*. Here, we have excluded the extraction of *hammers* to maintain consistency with the dataset standard [14] and the CHR [14] framework. From Table 5, we can see that although the proposed framework lags from [5]. However, because it utilizes meta-one-shot learning to recognize contraband items and still able to achieve comparable performance with the fully supervised frameworks (trained on the large-scale datasets), we believe that the performance of the proposed framework is promising. In addition, it should be noted that the comparison of the proposed framework with second-best [51] is not fair because they only studied SIXray10 [14] subset of the SIXray dataset [14] in their study for extracting only the *guns* and *knives*.

**Figure 5:**
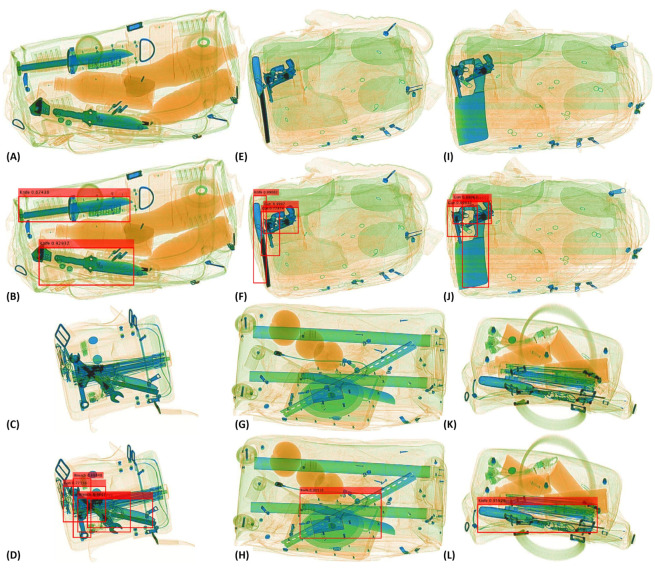
Qualitative performance of the proposed framework on the SIXray [14] dataset for detecting contraband items such as *knives*, *guns*, and *wrenches* as shown in (**A**–**L**). Moreover, first and third row show the original scans. These samples showcase the effectiveness of the proposed framework in detecting *gun*, *knife*, and *wrenches* in (**D**), a cluttered *knife* in (**H**,**L**), and the overlapping *guns* and a *knife* in (**J**).

Apart from this, the capacity of the proposed framework to localize and detect the baggage threat can be seen in Figure 5. Here, we can observe how remarkably the extremely cluttered contraband items have been detected, e.g., see the detected *gun*, *knife*, and *wrenches* in (D), a cluttered *knife* in (H and L), the overlapping *guns* and a *knife* in (J). This is due to the fact that the proposed tensor-shot detector can suppress the unwanted edges while emphasizing the threatening regions within the candidate scan through its backbone.

### 6.4. Qualitative Analysis

**Figure 6:**
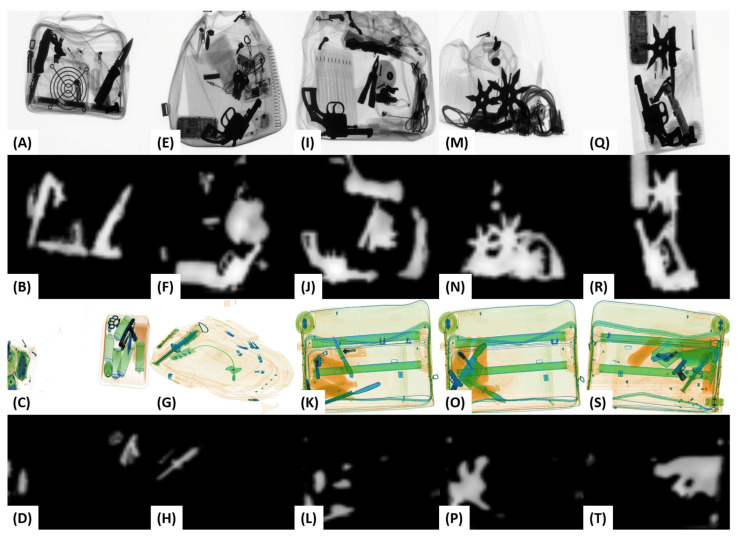
Saliency maps produced by the proposed salient feature extractor on both GDXray [15] and SIXray [14] datasets are shown in (**A**–**T**). Also, the first and third row show the original scans.

**Figure 7:**
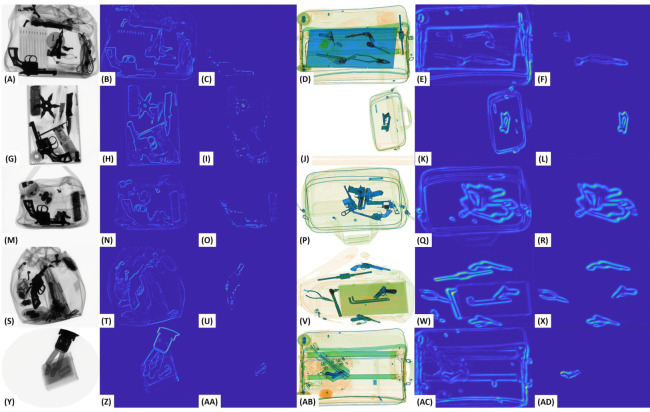
Irrelevant edge suppression through the proposed meta-transfer learning-driven tensor-shot detection framework are shown in ((**A**)–(**AD**)). Also, the first and fourth column show the original scans.

Figure 6 shows the saliency maps obtained from the proposed salient network. Although, due to its shallow architecture, the salient model cannot generalize well against the diverse ranging X-ray scanners. Nevertheless, it can robustly pick the high transitional objects and suppress them for generating the low-energy tensors, e.g., see the extracted *knives* and *guns* in Figure 6 (L, P, and T) despite the extreme clutter. Moreover, the proposed dual-energy tensor scheme can reveal the boundaries of the low and high spectral threatening items; it also amplifies the transitions of normal baggage content (e.g., see the second and fifth columns in Figure 7). Here, to suppress the irrelevant edges, we employ a meta-transfer learning-driven backbone network (as discussed in Section 4.5) that is trained on the large-scale generalized dual-tensor representations of the grayscale and color X-ray scans. Furthermore, this backbone network is fine-tuned via single-shot training to recognize different contraband item proposals. The suppressed edges (computed by the generalized backbone model) can be seen in Figure 7. Here, we can appreciate its capacity to effectively strain the irrelevant edges regardless of the scanner specifications. Although, compared to GDXray [15], the backbone model produces better edge representations for the SIXray [14] dataset scans. This is because the backbone network is more biased towards SIXray [14] scanner as compared to the GDXray [14] due to the imbalanced ratio of the training scans within both datasets. However, this situation can be easily handled by employing different binarization thresholds (for each dataset) during postprocessing.

**Figure 8:**
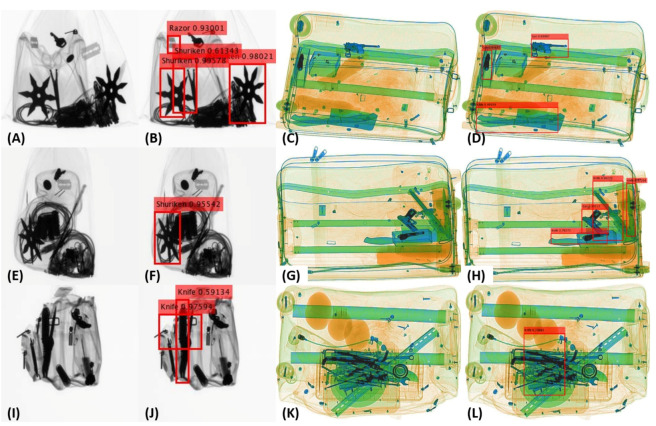
Failure cases on the GDXray [15] and SIXray [14] dataset are shown in (**A**–**L**). Moreover, the first and third column show the original scans.

Despite the weak edge representations obtained for the GDXray [15] scans, the capacity of the generalized backbone model for edge suppression can be appreciated in Figure 7 (AA), where it has effectively retained the *razor* while suppressing all the irrelevant edges regardless of their prominence in the scan. Moreover, in Figure 8, we report some of the failures cases of the proposed tensor-shot detector on both datasets. The first failure is related to the incapacity of the edge suppression backbone to eliminate the irrelevant boundaries of the baggage content that produces multiple bounding boxes for the same item e.g., see the twice detected *shuriken* in (B). Although, we handled such failures by applying nonmaximum suppression [55] as a postprocessing step. Still, because of the fixed overlapping threshold (in the nonmaximum suppression [55]), we rarely observed these errors. Although, we can avoid them by further decreasing the overlapping threshold. In addition, the other failure related to nonmaximum suppression [55] is the generation of loose bounding boxes, e.g., see the bounding box of a cluttered *knife* in (J). These loose boxes are generated by merging the multiple overlapping boxes (representing the same item). Although such errors are not drastic (as the framework is correctly detecting the item), such loose boxes can lead towards a low quantitative performance when compared to the ground truth. Moreover, the proposed framework also misses some extremely dulled and occluded objects, e.g., the *razor* in (B and F) (also in Figure 4P), and a *gun* in (H). These types of failures are related to the inability of the saliency model to accurately differentiate the low contrasted (and overlapping) objects within the low-energy tensor. Although we observed very few of these cases, they can be easily addressed by amplifying the dual-energy tensors before edge suppression. The last failure which we observed is the inability of the proposed tensor-shot detector to accurately detect all the overlapping instances of the same time in extremely challenging scenario, e.g., see the missed *knife* on top of *chopper* in (D), and the missed wrenches in (L), even the bounding box of the detected *knife* is not accurate. While we concur that the proposed framework is limited towards these false negatives (only if the scans are extremely challenging like Figure 8C,K), we can still appreciate the overall detection performance of the proposed framework given the fact that it is highly generalizable, and yet, produces decent detection results (even in cluttered scenarios), e.g., see the detected *knife* in (L) and the cluttered *knife* in (D).

### 6.5. Generalizability Assessment

To further test the generalizability of the proposed tensor-shot detector, we conducted another experiment where we trained the edge suppression backbone network (ResNet-101 [46]) on the dual-tensor representations of the training scans (of both datasets) and utilized a zero-shot classifier (driven through the generalized backbone model) to classify the proposals of contraband items. The detection performance of the proposed framework for this experimentation is shown in Table 7. Here, we can see that the proposed zero-shot tensor-shot driven detector achieved an *mAP* score of 0.8069 on GDXray [15], and 0.4690 on SIXray [14] using ResNet-101 [46] as a backbone. In addition, the proposed framework achieved an *mAP* score of 0.6528, 0.4379, and 0.3164 on SIXray10, SIXray100, and SIXray1000 subset, respectively. Although, on average, the performance of the zero-shot detector lags by 11.50% on GDXray [15] and 27.36% on SIXray [14] dataset as compared to the one-shot detector but still the performance of the zero-shot detector is appreciable given the fact that the classifier does not require any fine-tuning even on single training examples.

## 7. Discussion and Conclusions

This paper presents a meta-transfer learning-based tensor-shot detection framework that can recognize highly concealed and cluttered baggage threats from the security X-ray scans. The proposed framework has been thoroughly tested on the two publicly available datasets (i.e., the SIXray [14] and the GDXray [15]). In addition, it has been extensively compared with the existing state-of-the-art solutions where it achieved 0.573% improvements (in terms of F_1_ score) over [33] on GDXray [15] dataset and 8.03% improvements (in terms of *mAP*) over [14] on the SIXray [14] dataset.

Furthermore, through both quantitative and qualitative evaluations, we have demonstrated the capacity of the proposed framework for detecting the extremely cluttered contraband items on both grayscale and colored X-ray scanners. For instance, see the extraction of cluttered (and occluded) *pistol* and *revolver* in Figure 4D,F,J,L. In addition, in Figure 5, see the extraction of extremely occluded *gun*, *wrench* and *knife* in (D), a *knife* in (H and L). Moreover, Table 4 and Table 6 further showcase the capacity of the proposed framework towards recognizing contraband items regardless of the occlusion, baggage clutter, and concealment.

Apart from this, the proposed framework is, to the best of our knowledge, the first baggage threat detector that is invariant to the scanner specifications and can work on any grayscale or colored X-ray scan for recognizing the potential threats. This is due to its capacity to transform the candidate scan into novel dual-energy tensors from which it identifies the threatening items even in extreme clutter and concealment. In addition, the proposed framework can be practically deployed in the real world for mass screening baggage threats (including the cluttered ones which, although, modern X-ray scanners can reveal, yet they can be missed by the security officers during the manual inspection due to the rush hours and tiring work schedule).

In future, the proposed tensor-shot framework can be utilized in detecting 3D printed and dismantle items from the baggage X-ray scans which are barely visible even to the human observers. Furthermore, it can also be tested on normal RGB scans for detecting concealed, cluttered, and occluded objects.

## Figures and Tables

**Table 1:** Summary of the state-of-the-art baggage threat detection frameworks *.

Literature	Methodology	Performance	Limitations
Miao et al. [14]	Developed CHR [14], an imbalanced resistant framework that leverages reversed connections class-balanced loss function to effectively learn the imbalanced suspicious item categories in a highly imbalanced SIXray [14] dataset.	Achieved an overall mean average precision score of 0.793, 0.606, and 0.381 on SIXray10, SIXray100, and SIXray1000 [14], respectively when coupled with ResNet-101 [46] for recognizing five suspicious item categories.	Although the framework is resistant to an imbalanced dataset, it is still tested only on a single dataset.
Hassan et al. [11]	Proposed a contour instance segmentation framework for recognizing baggage threats regardless of the scanner specifications.	Achieved a mean average precision score of 0.4657 on a total of 223,686 multivendor baggage X-ray scans.	Built upon a conventional fine-tuning approach that requires a large-scale training dataset.
Gaus et al. [51]	Evaluated the transferability of different one-staged and two-staged object detection and instance segmentation models on SIXray10 [14] subset of the SIXray [14] dataset and also on their locally prepared dataset.	Achieved a mean average precision of 0.8500 for extracting *guns* and *knives* on SIXray10 [14] dataset.	Tested on only one public dataset i.e., the SIXray10 [14] for only extracting *guns* and *knives*.
Wei et al. [13]	Proposed a plug-and-play module dubbed DOAM [13] that can be integrated with the deep object detectors to recognize and localized the occluded threatening items.	Achieved the mean average precision score of 0.740 coupled with SSD [54].	DOAM [13] is not tested on publicly available GDXray [15] and SIXray [14] datasets.
Hassan et al. [5]	Developed a CST framework that leverages contours of the baggage content to generate object proposals that are screened via a single classification backbone.	Achieved a mean average precision score of 0.9343 and 0.9595 on GDXray [15] and SIXray [14] datasets.	CST, although, is tested on two public datasets, but it requires extensive parameter tuning to work well on both of them.

* For a detailed overview on the existing approaches, we refer the reader to the Supplementary Material of this article.

**Table 2:** Effect of varying focal loss parameters on the detection performance (in terms of *mAP*) on GDXray [15] and SIXray [14] dataset. Bold indicates the optimal performance.

GDXray [15]		α		
		0.25	0.5	0.75
	1	0.9059	0.8742	0.8693
	2	**0.9162**	0.8916	0.8869
γ	3	0.9143	0.8882	0.8807
	4	0.9017	0.8834	0.8762
	5	0.9064	0.8763	0.8691
SIXray [14]		α		
		0.25	0.5	0.75
	1	0.5483	0.5140	0.4926
	2	**0.6457**	0.6283	0.6182
γ	3	0.6283	0.6036	0.5874
	4	0.6156	0.5709	0.5370
	5	0.6083	0.5472	0.5198

Scores for the SIXray dataset represent the average of SIXray10, SIXray100, and SIXray1000 subset.

**Table 3:** Performance of proposed detection framework in terms of *mAP* on GDXray [15] and SIXray [14] datasets using different classification backbones. Bold indicates the best performance.

Network	GDXray [15]	SIXray10 [14]	SIXray100 [14]	SIXray1000 [14]	SIXray [14] *
VGG-16 [48]	0.8691	0.7583	0.5721	0.4126	0.5810
ResNet-50 [46]	0.8917	0.7826	0.6284	0.4392	0.5915
ResNet-101 [46]	**0.9162**	**0.8053**	**0.6791**	**0.4527**	**0.6457**

* Average of SIXray10, SIXray100 and SIXray1000 subset.

**Table 4:** Detection performance of the proposed framework on the GDXray [15] and the SIXray [14] dataset. Bold indicates the best performance while the second-best performance is underlined. ’-’ denotes that the metric is not computed.

Dataset	Metric	Proposed	[14]	[51]	[33]	[31]	[65]	[66]
GDXray [15]	mean *IoU*	**0.9118**	-	-	-	-	-	-
	mean *DC*	**0.9536**	-	-	-	-	-	-
	*mAP*	**0.9162**	-	-	-	-	-	-
	Accuracy	0.9554	-	-	**0.9840**	0.9500	-	-
	*TPR*	0.9761	-	-	**0.9800**	-	0.8900	0.9430
	*TNR*	0.9305	-	-	-	0.9140	-	**0.9440**
	*FPR*	0.0694	-	-	-	0.0860	-	**0.0560**
	*PPV*	**0.9441**	-	-	0.9300	-	0.9200	-
	F_1_	**0.9598**	-	-	0.9543	-	0.9047	-
SIXray [14] *	mean *IoU*	**0.9238**	-	-	-	-	-	-
	mean *DC*	**0.9603**	-	-	-	-	-	-
	*mAP*	0.6457	0.5938	**0.8500**	-	-	-	-
	Accuracy	**0.8949**	0.4577	-	-	-
	*TPR*	**0.8127**	-	-	-	-
	*TNR*	**0.8956**	-	-	-	-
	*FPR*	**0.1043**	-	-	-	-
	*PPV*	**0.0621**	-	-	-	-
	F_1_	**0.1153**	-	-	-	-	-	-

* Average of SIXray10, SIXray100, and SIXray1000 subset.

**Table 5:** Statistical significance of the proposed framework compared to the state-of-the-art solutions in terms of *MSE*. Bold indicates the best results, while the second-best performances are underlined. ’-’ indicates that the metric is not computed. Scores for the SIXray dataset represent the average of SIXray10, SIXray100, and SIXray1000 subset.

Dataset	Items	Proposed	[14]	[51]	[5]
GDXray [15]	Handguns	**0.001436**	-	-	0.008082
	Knives	0.002683	-	-	**0.000030**
	Razors	**0.007586**	-	-	0.013782
	Shuriken	0.001459	-	-	**0.000068**
	Mean	**0.003291**	-	-	0.005490
	STD	**0.002530**	-	-	0.005802
SIXray [14]	Guns	0.021874	0.018496	0.006400	**0.000079**
	Knives	0.030905	0.021432	0.044100	**0.004264**
	Wrenches	0.060614	0.101251	-	**0.000072**
	Scissors	0.134762	0.166219	-	**0.000038**
	Pliers	0.087971	0.030241	-	**0.005372**
	Mean	0.067225	0.067528	0.025250 *	**0.001965**
	STD	0.041015	0.057959	0.018850 *	**0.002355**

* These results are computed by only considering *guns* and *knives* items from SIXray10 [14] subset.

**Table 6:** Quantitative evaluation of the proposed framework and other state-of-the-art frameworks on SIXray [14] dataset towards detecting contraband items exhibiting different level of clutter and concealment.

Level of Clutter and Concealment	Proposed	
Low	0.7816	0.7453
Partial or mild	0.6593	0.5918
Full or extreme	0.5201	0.4632

**Table 7:** Detection performance of a zero-shot classifier (in terms of *mAP*) driven through the generalized edge suppression backbone.

Network	GDXray [15]	SIXray10 [14]	SIXray100 [14]	SIXray1000 [14]	SIXray [14]
ResNet-101 [46]	0.8069	0.6528	0.4379	0.3164	0.4690

## Supplementary Materials

The following are available online at https://www.mdpi.com/1424-8220/20/22/6450/s1, Table S1. Summary of existing works related to autonomous baggage threat detection.
