# Supplementary material for: Meta-Transfer Learning Driven Tensor-Shot Detector for the Autonomous Localization and Recognition of Concealed Baggage Threats
Source: Sensors (Basel). 2020 Nov 12;20(22):6450. doi: 10.3390/s20226450 (PMC7696514; doi:10.3390/s20226450)
Supplement: Supplementary file 1 [file sensors-20-06450-s001.pdf]

Meta-Transfer Learning Driven Tensor-Shot Detector for the Autonomous Localization and Recognition of Concealed Baggage Threats

Supplementary Material

This supplementary material reports a detailed summary of the related work discussed in Section 2 of the article.

Table S1. Summary of existing works related to autonomous baggage threat detection.

Literature	Methodology	Performance	Limitations
Turcsany et al. [1]	Proposed SURF coupled SVM model driven through Bag of Words to classify baggage threats.	Achieved a true positive rate of 0.9907 and a false positive rate of 0.0431 on a local dataset.	The framework can classify scans containing suspicious items but does not possess the capacity to localize them. In addition, it is tested in limited experimental settings.
Heitz et al. [2]	Developed a framework dubbed SATISφ for segmenting objects within X-ray imagery using additivity in log space.	Achieved the root-mean-squared error of 0.66.	SATISφ is applied only to a limited set of X-ray scans.
Bastan [3]	Developed dense sampling and multiview branch and bound search scheme to detect objects within texture-less X-ray scans.	Achieved an average precision score of 0.704, 0.851, 0.259 for detecting *laptops*, *bottles*, and *handguns*, respectively, using the custom prepared dataset.	The framework presented in this paper is vulnerable to diverse ranging scanners.
Bastan et al. [4]	Used Bag of Visual Words with different feature descriptors to recognize baggage threats within X-ray imagery.	Achieved the average precision score of 0.38, 0.43, and 0.39 for recognizing threatening items within low-energy, high-energy, and color X-ray scans, respectively.	This framework is validated on a limited set of locally acquired scans.
Kundegorski et al. [5]	Coupled feature descriptors with Bag of Words, SVM, and Random Forest model for classifying baggage X-ray scans containing prohibited items.	Achieved the optimal accuracy of 0.94 for recognizing firearms scans via FAST-SURF.	This framework is tested only on a local dataset under a constraint environment.

Table S1. *Cont.*

Mery et al. [6]	Proposed an object detector dubbed Adaptive Sparse Representation (XASR+) that is trained patch-wise forming a representative set of dictionaries for each object. During test time, the random patches (from the test scans) are generated forming the "best" dictionary (of each object) through which the candidate test patch is classified via the Sparse Representation Classification (SRC) scheme.	XASR+ achieved an accuracy of more than 0.95 (and 0.85 for 15% occluded objects).	XASR+ is tested under a very constrained environment.
Riffo et al. [7]	Developed an AISM framework that uses a visual vocabulary driven via occurrence structure to detect threatening objects within X-ray imagery.	AISM is tested on GDXray [8] dataset and achieved the recall and false positive rate of 0.97 and 0.06 for *shuriken*, 0.99 and 0.02 for detecting *razors*, and 0.89 and 0.18 for detecting *handguns*.	AISM cannot well generalize to multiple scanner specifications.
Liu et al. [9]	Used YOLO9000 [10] to detect contraband items from security X-ray scans.	YOLO9000 [10] achieved the average precision and recall rate of 0.945 and 0.926, respectively.	YOLO9000 [10], in this paper, is tested only on locally acquired 2850 scans for detecting *scissors* and *aerosols*.
Dhiraj et al. [11]	Evaluated Faster RCNN [12], YOLOv2 [10], and Tiny YOLO [10] for detecting threatening items from baggage X-ray scans.	Achieved the accuracy, recall, and precision score of 0.9840, 0.9800, and 0.9300 on the GDXray [8] dataset.	All the one-staged and two-staged detectors (used in this study) are tested only on the GDXray [8] dataset.
Xu et al. [13]	Enhanced the capacity of the CNN model to localize threatening items via attention mechanism.	Achieved the recognition and localization accuracy of 0.956, 0.53 for detecting *revolvers*, 0.983 and 0.735 for detecting *guns*, and 0.972 and 0.541 for detecting *knives*.	This study only involved one public dataset, i.e., the GDXray [8] dataset.
Jaccard et al. [14]	Used sliding-window CNN models to detect *cars* within cargo transmission X-ray imagery.	Achieved classification rate of 100% for recognizing *cars* for a false positive rate of 1-in-454.	Sliding-window CNN is tested for only binary classification (*cars* versus *no-cars*) tasks on a limited dataset.

Griffin et al. [15]	Detected threatening baggage items as anomalies based upon their shape, density, and textural representations.	Recognizing 90% of firearms as anomalies while achieving the false positive rate of 0.18.	The presented framework cannot localize the recognized anomalous items.
An et al. [16]	Developed a semantic segmentation model-driven via a dual-attention mechanism to detect contraband items within security X-ray scans.	Achieved a mean IoU score of 0.683 for extracting prohibited items.	The semantic segmentation model proposed in this paper is tested only on a locally acquired dataset for extracting baggage threats with no or low occlusion.
Hassan et al. [17]	Developed a contour instance segmentation framework for recognizing baggage threats regardless of the scanner specifications.	Achieved a mean average precision score of 0.4657 on a total of 223,686 multivendor baggage X-ray scans.	The contour instance segmentation framework proposed in this paper is built upon a conventional fine-tuning approach that requires a large-scale training dataset.
Zou et al. [18]	Used sampling contours extracted via variable thresholds to detect suspicious items within security X-ray scans.	Achieved a mean average precision of 0.864 and a recall rate of 0.877.	The framework is tested only on a custom prepared grayscale dataset.
Akcay et al. [19]	Used AlexNet [20] as a feature extractor coupled with SVM for classifying baggage threats. Furthermore, the authors compared Faster R-CNN [12], sliding-window based CNN (SW-CNN), region-based fully convolutional networks (R-FCN) [21], and YOLOv2 [10] for detected occluded contraband items from the X-ray imagery.	Achieved a mean average precision score of 0.885 using YOLOv2 with input size 544×544. Using the input of size 416×416, the authors achieved a mean average precision score of 0.974 for two-class firearm detection.	This study is conducted using only locally prepared datasets.
Xiao et al. [22]	Developed a computationally efficient variant of Faster R-CNN [12] dubbed R-PCNN for detecting prohibited items from THz imagery.	Achieved the detection accuracy of 0.845 with an average detection time of 20 milliseconds.	R-PCNN is tested in a constraint environment on locally acquired THz scans.

Gaus et al. [23]	Evaluated RetinaNet [24], Faster R-CNN [12], and Mask R-CNN [25] for screening baggage X-ray scans as benign or malignant.	Achieved the mean average precision of up to 0.979 for six-class detection and also achieved the accuracy of 0.66 for anomaly identification.	This study only used locally prepared (private) datasets.
Wei et al. [26]	Proposed a plug-and-play module dubbed DOAM [26] that can be integrated with the deep object detectors to recognize and localized the occluded threatening items.	Achieved the mean average precision score of 0.740 when coupled with SSD [27].	DOAM has not been tested on publicly available GDXray [8] and SIXray [28] datasets.
Miao et al. [28]	Developed CHR [28], an imbalanced resistant framework that leverages reversed connections to derive high-level visual cues that aid in producing distinct mid-level features. Furthermore, the framework is trained via a custom class-balanced loss function to effectively learn the imbalanced suspicious item categories in a highly imbalanced SIXray [28] dataset.	Achieved an overall mean average precision score of 0.793, 0.606, and 0.381 on SIXray10 [28], SIXray100 [28], and SIXray1000 [28], respectively when coupled with ResNet-101 [29] for recognizing five suspicious item categories.	Although the framework is resistant to an imbalanced dataset, it is only tested on a single dataset.
Gaus et al. [30]	Evaluated the transferability of different one-staged and two-staged object detection and instance segmentation models on SIXray10 [28] subset of the SIXray [28] dataset and also on their locally prepared dataset.	Achieved a mean average precision of 0.8500 for extracting *guns* and *knives* on the SIXray10 [28] dataset.	This study involved only one public dataset, i.e., the SIXray10 [28] subset of the SIXray [28] dataset for extracting *guns* and *knives* only.
Hassan et al. [31]	Developed a CST framework that leverages contours of the baggage content to generate object proposals that are screened via a single classification backbone.	Achieved a mean average precision score of 0.9343 and 0.9595 on GDXray [8] and SIXray [28] datasets.	Although the CST framework is resistant to imbalanced data and is tested on two public datasets. However, it requires extensive parameter tuning to work well on each dataset.

Table S1. *Cont.*

Akcay et al. [32]	Detected baggage threats as anomalies via unsupervised adversarial learning-driven encoder-decoder-encoder topology dubbed GANomaly [32].	Achieved area-under-the-curve (AUC) score of 0.643 and 0.882 on local baggage X-ray datasets.	GANomaly [32] is computationally expensive because of an additional encoder block.
Akcay et al. [33]	Modified GANomaly [32] by adding skip-connections between encoder-decoder networks and eliminating the redundant encoder backbone.	Achieved an AUC score of 0.940 and 0.903 on the two local datasets. In addition, Skip-GANomaly [33] achieved an AUC score of 0.953 for detecting *cars* on CIFAR-10.	Skip-GANomaly [33] is not tested for reconstructing high-resolution baggage X-ray scans to detect threatening anomalous items.

References

1. Turcsany, D.; Mouton, A.; Breckon, T.P. Improving Feature-based Object Recognition for X-ray Baggage Security Screening using Primed Visual Words. In Proceedings of the 2013 IEEE International Conference on Industrial Technology (ICIT), Cape Town, South Africa, 25–28 February 2013; pp. 1140–1145.
2. Heitz, G.; Chechik, G. Object Separation in X-ray Image Sets. In Proceedings of the IEEE International Conference on Computer Vision and Pattern Recognition (CVPR), San Francisco, CA, USA, 13–18 June 2010; pp. 2093–2100.
3. Baştan, M. Multi-view Object Detection In Dual-energy X-ray Images. *Mach. Vis. Appl.* **2015**, 26, 1045–1060.
4. Baştan, M.; Yousefi, M.R.; Breuel, T.M. Visual Words on Baggage X-ray Images. In Proceedings of the 14th International Conference on Computer Analysis of Images and Patterns, Seville, Spain, 29–31 August 2011; pp. 360–368.
5. Kundegorski, M.E.; Akçay, S.; Devereux, M.; Mouton, A.; Breckons, T.P. On using Feature Descriptors as Visual Words for Object Detection within X-ray Baggage Security Screening. In Proceedings of the IEEE International Conference on Imaging for Crime Detection and Prevention (ICDP), Madrid, Spain, 23–25 November 2016; pp. 1–6.
6. Mery, D.; Svec, E.; Arias, M. Object Recognition in Baggage Inspection Using Adaptive Sparse Representations of X-ray Images. In *Pacific-Rim Symposium on Image and Video Technology*; Springer: Cham, Switzerland, 2016; pp. 709–720.
7. Riffo, V.; Mery, D. Automated Detection of Threat Objects Using Adapted Implicit Shape Model. *IEEE Trans. Syst. Man Cybern. Syst.* **2016**, 46, 472–482.
8. Mery, D.; Riffo, V.; Zscherpel, U.; Mondragón, G.; Lillo, I.; Zuccar, I.; Lobel, H.; Carrasco, M. GDXray: The database of X-ray images for nondestructive testing. *J. Nondestruct. Eval.* **2015**, 34, 42.
9. Liu, Z.; Li, J.; Shu, Y.; Zhang, D. Detection and Recognition of Security Detection Object Based on YOLO9000. In Proceedings of the 2018 5th International Conference on Systems and Informatics (ICSAI), Nanjing, China, 10–12 November 2018; pp. 278–282.
10. Redmon, J.; Farhadi, A. YOLO9000: Better, Faster, Stronger. In Proceedings of the IEEE International Conference on Computer Vision and Pattern Recognition (CVPR), Honolulu, HI, USA, 21–26 July 2017; pp. 7263–7271.
11. Jain, D.K. An evaluation of deep learning based object detection strategies for threat object detection in baggage security imagery. *Pattern Recognit. Lett.* **2019**, 120, 112–119.
12. Ren, S.; He, K.; Girshick, R.; Sun, J. Faster R-CNN: Towards Real-Time Object Detection with Region Proposal Networks. In Proceedings of the 29th Conference on Neural Information Processing Systems (NIPS 2015), Montreal, Canada, 7–12 December 2015; pp. 91–99.

13. Xu, M.; Zhang, H.; Yang, J. Prohibited Item Detection in Airport X-Ray Security Images via Attention Mechanism Based CNN. In Proceedings of the Chinese Conference on Pattern Recognition and Computer Vision, Guangzhou, China, 23–26 November 2018; pp. 429–439.
14. Jaccard, N.; Rogers, T.W.; Morton, E.; Griffin, L.D. Detection of Concealed Cars In Complex Cargo X-ray Imagery Using Deep Learning. J. *X-Ray Sci. Technol*. **2017**, 25, 323–339.
15. Griffin, L.D.; Caldwell, M.; Andrews, J.T.A.; Bohler, H. "Unexpected Item in the Bagging Area": Anomaly Detection in X-Ray Security Images. *IEEE Trans. Inf. Forensics Secur.* **2019**, 14, 1539–1553.
16. An, J.; Zhang, H.; Zhu, Y.; Yang, J. Semantic Segmentation for Prohibited Items in Baggage Inspection. In Proceedings of the International Conference on Intelligence Science and Big Data Engineering Visual Data Engineering, Nanjing, China, 17–20 October 2019; pp. 495–505.
17. Hassan, T.; Akçay, S.; Bennamoun, M.; Khan, S.;Werghi, N. Trainable Structure Tensors for Autonomous Baggage Threat Detection Under Extreme Occlusion. *arXiv* **2020**, arXiv:2009.13158.
18. Zou, L.; Yusuke, T.; Hitoshi, I. Dangerous Objects Detection of X-Ray Images Using Convolution Neural Network. In *Security with Intelligent Computing and Big-data Services*; Springer: Cham, Switzerland, 2018.
19. Akçay, S.; Kundegorski, M.E.; Willcocks, C.G.; Breckon, T.P. Using Deep Convolutional Neural Network Architectures for Object Classification and Detection Within X-ray Baggage Security Imagery. *IEEE Trans. Inf. Forensics Secur*. **2018**, 13, 2203–2215.
20. Krizhevsky, A.; Sutskever, I.; Hinton, G.E. ImageNet Classification with Deep Convolutional Neural Networks. In Proceedings of the 26th Annual Conference on Neural Information Processing Systems (NIPS 2012), Lake Tahoe, NV, USA, 3–8 December 2012; pp. 1106–1114.
21. Dai, J.; Li, Y.; He, K.; Sun, J. R-FCN: Object Detection via Region-based Fully Convolutional Networks. In Proceedings of the 30th Conference on Neural Information Processing Systems (NIPS 2016), Barcelona, Spain, 5–10 December 2016; pp. 379–387.
22. Xiao, H.; Zhu, F.; Zhang, R.; Cheng, Z.; Wang, H.; Alesund, N.; Dai, H.; Zhou, Y. R-PCNN Method to Rapidly Detect Objects on THz Images in Human Body Security Checks. In Proceedings of the IEEE SmartWorld, Ubiquitous Intelligence & Computing, Advanced & Trusted Computing, Scalable Computing & Communications, Cloud & Big Data Computing, Internet of People and Smart City Innovation, Guangzhou, China, 8–12 October 2018; pp. 1777–1782.
23. Gaus, Y.F.A.; Bhowmik, N.; Akçay, S.; Guillén-Garcia, P.M.; Barker, J.W.; Breckon, T.P. Evaluation of a Dual Convolutional Neural Network Architecture for Object-wise Anomaly Detection in Cluttered X-ray Security Imagery. In Proceedings of the 2019 International Joint Conference on Neural Networks (IJCNN), Budapest, Hungary, 14–19 July 2019; pp. 1–8.
24. Lin, T.Y.; Goyal, P.; Girshick, R.; He, K.; Dollar, P. Focal Loss for Dense Object Detection. In Proceedings of the IEEE International Conference on Computer Vision and Pattern Recognition (CVPR), Honolulu, HI, USA, 21–26 July 2017; pp. 2980–2988.
25. He, K.; Gkioxari, G.; Dollár, P.; Girshick, R. Mask R-CNN. In Proceedings of the IEEE International Conference on Computer Vision (ICCV), Venice, Italy, 22–29 October 2017; pp. 2961–2969.
26. Wei, Y.; Tao, R.;Wu, Z.; Ma, Y.; Zhang, L.; Liu, X. Occluded Prohibited Items Detection: An X-ray Security Inspection Benchmark and De-occlusion Attention Module. *arXiv* **2020**, arXiv:2004.08656.
27. Liu, W.; Anguelov, D.; Erhan, D.; Szegedy, C.; Reed, S.; Fu, C.Y.; Berg, A.C. SSD: Single Shot MultiBox Detector. In Proceedings of the European Conference on Computer Vision, Amsterdam, The Netherlands, 8–16 October 2016; pp. 21–37.
28. Miao, C.; Xie, L.; Wan, F.; Su, C.; Liu, H.; Jiao, J.; Ye, Q. SIXray: A Large-scale Security Inspection X-ray Benchmark for Prohibited Item Discovery in Overlapping Images. In Proceedings of the IEEE International Conference on Computer Vision and Pattern Recognition (CVPR), Long Beach, CA, USA, 18–20 June 2019; pp. 2119–2128.
29. He, K.; Zhang, X.; Ren, S.; Sun, J. Deep Residual Learning for Image Recognition. In Proceedings of the IEEE International Conference on Computer Vision and Pattern Recognition (CVPR), Las Vegas, NV, USA, 26 June–1 July 2016; pp. 770–778.

30. Gaus, Y.F.A.; Bhowmik, N.; Akcay, S.; Breckon, T. Evaluating the Transferability and Adversarial Discrimination of Convolutional Neural Networks for Threat Object Detection and Classification within X-Ray Security Imagery. *arXiv* **2019**, arXiv:1911.08966.
31. Hassan, T.; Bettayeb, M.; Akçay, S.; Khan, S.; Bennamoun, M.;Werghi, N. Detecting Prohibited Items in X-ray Images: A Contour Proposal Learning Approach. In Proceedings of the 27th IEEE International Conference on Image Processing (ICIP), Abu Dhabi, UAE, 25–28 October 2020; pp. 2016–2020.
32. Akçay, S.; Atapour-Abarghouei, A.; Breckon, T.P. GANomaly: Semi-Supervised Anomaly Detection via Adversarial Training. In *Asian Conference on Computer Vision*; Springer: Berlin/Heidelberg, Germany, 2018; pp. 622–637.
33. Akçay, S.; Atapour-Abarghouei, A.; Breckon, T.P. Skip-GANomaly: Skip Connected and Adversarially Trained Encoder-Decoder Anomaly Detection. *arXiv* **2019**, arXiv:1901.08954.

© 2020 by the authors. Submitted for possible open access publication under the terms and conditions of the Creative Commons Attribution (CC BY) license (http://creativecommons.org/licenses/by/4.0/).
